# Analyzing Land Use Changes in the Metropolitan Jilin City of Northeastern China Using Remote Sensing and GIS

**DOI:** 10.3390/s8095449

**Published:** 2008-09-03

**Authors:** Dan Hu, Guodong Yang, Qiong Wu, Hongqing Li, Xusheng Liu, Xuefeng Niu, Zhiheng Wang, Qiong Wang

**Affiliations:** 1 State Key Laboratory of Urban & Regional Ecology, Research Center for Eco-Environmental Sciences, Chinese Academy of Sciences, P.O. Box 2871, Beijing 100085, P.R. China; E-mail: hudan@rcees.ac.cn (D. H.); 2 College of Geo-exploring Science and Technology, Jilin University, 6 West Minzhu Street, Changchun 130026, P.R. China; E-mails: wuqiong@jlu.edu.cn (Q. W.); lihq@jlu.edu.cn (H. L.); niuxf@jlu.edu.cn (X. N.); wzh19831221@sina.com (Z. W.); wqjlcy@163.com (Q. W.); Tel.: +86-431-87627036. Fax: +86-431-87627036; 3 The 3rd Surveying and Mapping Institute, Heilongjiang Bureau of Surveying and Mapping, 2 Cehui Road, Harbin 150086, P.R. China; E-mail: bluelifeddt@163.com (Xusheng Liu); Tel.: +86-451-86684898; Fax: +86-451-86665376

**Keywords:** Land use change, land use patterns, urban growth, remote sensing, GIS, Jilin City

## Abstract

Remote sensing and GIS have been widely employed to study temporal and spatial urban land use changes in southern and southeastern China. However, few studies have been conducted in northeastern regions. This study analyzed land use change and spatial patterns of urban expansion in the metropolitan area of Jilin City, located on the extension of Changbai Mountain, based on aerial photos from 1989 and 2005 Spot images. The results indicated that urban land and transportation land increased dramatically (by 94.04% and 211.20%, respectively); isolated industrial and mining land decreased moderately (by 29.54%); rural residential land increased moderately (by 26.48%); dry land and paddy fields increased slightly (by 15.68% and 11.78%, respectively); forest and orchards decreased slightly (by 5.27% and 4.61%, respectively); grasslands and unused land decreased dramatically (by 99.12% and 86.04%, respectively). Sloped dry land (more than 4 degrees) was mainly distributed on the land below 10 degrees with an east, southeastern and south sunny direction aspect, and most sloped dry land transformed to forest was located on an east aspect lower than 12 degrees, while forest changed to dry land were mainly distributed on east and south aspects lower than 10 degrees. A spatial dependency analysis of land use change showed that the increased urban land was a logarithmic function of distance to the Songhua River. This study also provided some data with spatial details about the uneven land development in the upstream areas of Songhua River basin.

## 1. Introduction

Land use and land cover change is a major variable of global change that affects ecological systems [1]. The impact of land use change to local ecosystems includes water contamination [2-8], air pollution [9], local climate change [9, 10] or biodiversity loss [11]. Urban expansion is of considerable interest for the study of land use change [12-16]. China has undergone an unprecedented rate and scale of land use change since the 1980's [17]. The study of land use change within urban areas in China has been mainly focused on the southern [18-21] or southeastern coastal regions [22-29], where both the level and speed of economic development are much higher than the northeastern areas. There is a lack of study of the magnitude, pattern and type of land use change in northeastern cities, and of the discussion on land use change differences between southern and northeastern China.

During the last two decades, the Perl and Yangzi deltas and the Bejing-Tianjing zone have become the three most outstanding economic regions in China. Although economic growth in the northeastern provinces is also relatively rapid, the total increase lags far behind the other three economic regions. As a tightly related economic region, the northeastern three provinces are one of the regions in China with intensive industrial and urbanizing activities. The economic development in the regions slowed down after 1990s due to the policy of planning economy and the level of land development and urbanization is also relatively lower than the southern regions in China. In 2003, the State Council initiated its Strategy of Revitalizing Northeast China and Other Old Industrial Bases, and four economical circles were brought into the strategy, including Harbin-Daqing-Qiqihaer economical circle, Changchun-Jilin economical circle, Shenyang-Central Liaoning economical circle and Dalian-Inshore economical circle [30]. The economic development has speeded up, and land development and urbanization have been strengthened since the initiation of the revitalizing strategy [30].

The northeastern three provinces have an area of 235 thousand km^2^ of cultivated land, and 40% of them are slope land. The slope degrees of these cultivated lands are mainly between 3 and 10 degrees, and those are also the areas with the most serious water and soil losses. Soil and water loss are extremely serious at the cultivated land distributed at the low mountain and hill areas under the deforestation [31]. China suffered a large number of countrywide flood disasters in 1998, and the central government established a strict policy for Reconverting Cultivated Land into Forest to protect the soil and water resources on the upstream lands of the major rivers all over the country, including the Songhua River. The extension of Changbai Mountain area is one of the major regions with large areas of slope cultivated land, which is also confronted with the soil and water loss. There is an urgent need to study land use transformation to verify the effectiveness of the policy of Reconverting Cultivated Land into Forest in the upstream area of Songhua River.

Remote sensing has been widely used to analyze and model land use change [14, 17, 19, 22-25, 32-40], while geographic information systems (GIS) are a flexible platform for data storage, management and analysis [13, 22, 25, 37, 38]. The analysis of the pattern of urban growth provides information for the study of urban morphology, which has become a central element for urban sustainability [25].

This study analyzed the amount and type of land use change between 1989 and 2005, in the metropolitan area of Jilin City, Jilin Province. Land use change between dry land and forest on slope land over 4 degrees were analyzed. Buffer zone analysis and regression analysis were applied to identify the spatial dependency of urban expansion. The difference of land use change between southern coastal China and northeastern China was discussed. Remote sensing and global positioning system (GPS) were applied to quantify and analyze land use change.

## 2. Study area

**Figure 1.**
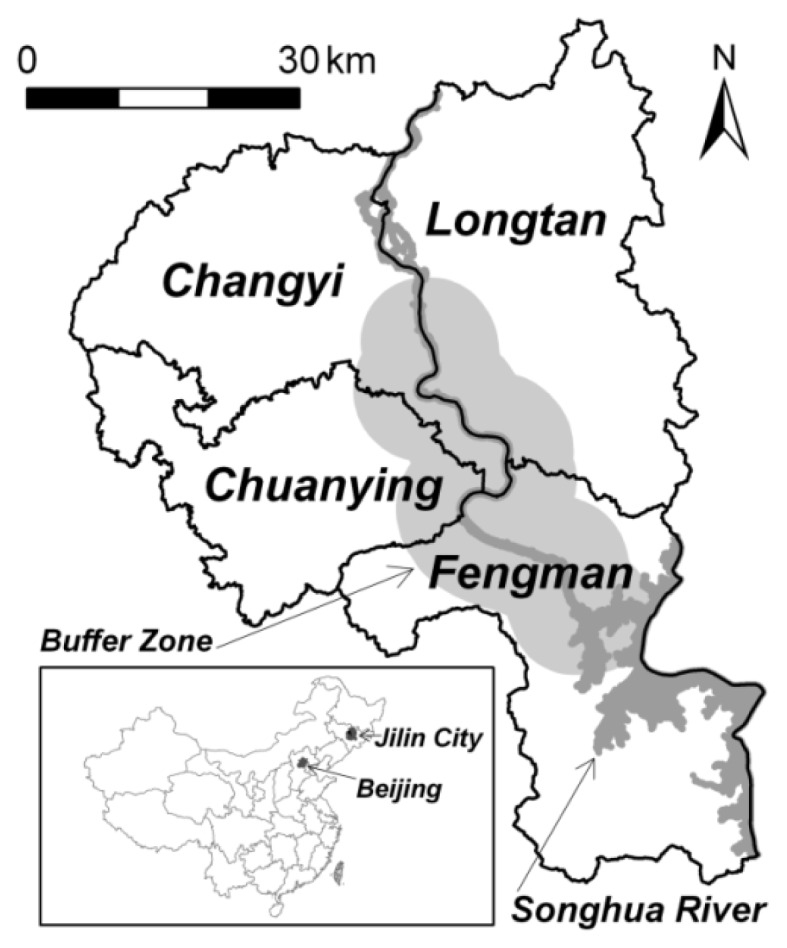
A map of the study area: the four districts of the metropolitan Jilin City and the buffer zone.

Jilin City is located at the transitional zone areas from Changbai Mountain Range to Songliao Plain with latitudes 42°31′N and 44°40′N, and longitudes 125°40′E and 127°56′E, and has an area of about 3,775 km^2^ (Figure 1). Changbai Mountain Range is the source of Songhua River, which flows northward out of the Changbai Mountains and cuts across the Manchurian Plain before emptying into the Heilongjiang River that separates northeastern China from Russia's Far East, the River continues into the Russian Federation and is there named the Amur River. The topography of Jilin City is characterized as gradually sloping down from southeast to northwest, and the metropolitan area is situated along the Songhua River's upstream, with Songhua Lake to the south. The average annual temperature is about 4°C, and the average precipitation is about 650 mm.

**Figure 2.**
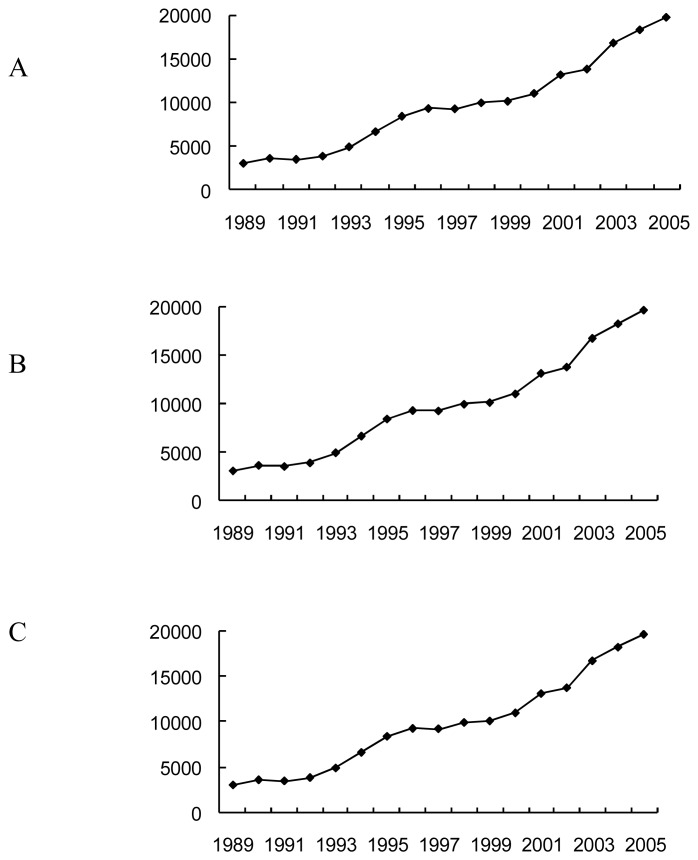
Major social economic indicators in the study area. A: Population increase (Tens of thousands); B: GDP increase (Million Yuan, RMB); C: Per capita GDP (Yuan, RMB)

Some major social economic indicators are shown in Figure 2. Jilin City has one of the largest petrochemical industry bases in China. The chemical industries have been constructed since the 1960s, and all of the petrochemical industries and related industries are currently integrated into the Jilin Petrochemical Company under the China National Petroleum Corporation. The petrochemical industry is still expanding and has become the largest industry in Jilin City. Like other urban areas in China, the metropolitan areas of Jilin City have experienced rapid urbanization during last two decades, such as population growth, economic growth and landscape alteration, which puts the Songhua River at high risk of chemical pollution and the land use and land planning in Jilin City are of extreme importance for the conservation of the river ecosystem.

## 3. Methods

### 3.1. Change detection

Land use patterns for 1989 (1:10,000) were mapped based on scanned natural color aerial photos with 1 m pixel size; land use patterns for 2005 (1:10,000) were mapped based on Spot 5 images. Both types of images were orthorectified to the national geodetic coordinate system using GPS (global poisoning system) ground control points, DEM models (1:10,000) and topographical maps (1:10,000), and the average location errors of each image were less than 5 m. In order to guarantee the consistency and accuracy of data processing, visual interpretation was applied for the land use classification. The classification schemes include: (1) urban (URB), (2) isolated industrial or mining land out of urban areas (IIM), (3) rural residence (RR), (4) transportation (TRN), (5) dry land (DL), (6) paddy field (PAD), (7) forest (FOR), (8) orchard (ORC), (9) grassland (GRS), (10) water (WAT), (11) unused (UNE). The land use classification for 1989 was corrected for errors using field surveys and land use map for each district, town and village with a scale of 1:10,000; the land use map for 2005 were corrected patch by patch through field survey using GPS for the redefinition of patch boundaries. A total of 20% land use patches were selected randomly to calculate the classification accuracy, and the total accuracy is higher than 0.95 for each of the two date land use maps. Slope map of the study area was created based on the 1:10,000 topology maps.

### 3.2. Change between forest and dry land on slope land

Of the 3,774.62 km^2^ study area, sloped land over 4 degrees occupied 953.22 km^2^. Land use changes between dry land and forest were the major land use transformation phenomena on sloped lands over 4 degrees. Overlay analysis was performed between the slope map and land use map of the two periods. Dry land and forest land and the transformation between these two types of maps were also analyzed.

### 3.3. Analysis of spatial dependency of urban expansion

Urban expansion is hypothesized as a function of distance to the Songhua River; that is, the increased urban land may be in a distance decay function. Regression analysis can be undertaken to verify this hypothesis. A series of buffer rings with a buffer distance of 0.1 km were created outside of the Songhua River within the central urban area of Jilin City (Figure 1). The relationship between the increased urban land and the distance to Songhua River is formulated by the following logarithmic model:

$$\text{URBincreased}=a_{0}+(a_{1}*\ln\;(x))$$

where *URBincreased* is the increased urban land (in ha) in the buffer rings, *a_0_* and *a_1_* are the coefficients, and *x* (in km) is the distance of the corresponding buffer ring to the Songhua River.

## 4. Results

### 4.1. Land use change in the metropolitan area of Jilin City

**Figure 3.**
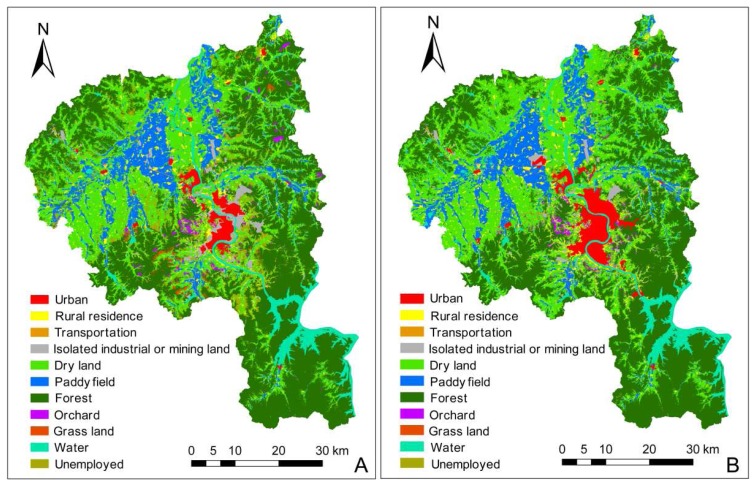
Land use maps in 1989 (A) and 2005 (B) in metropolitan Jilin City

Land use changes in each category in the whole metropolitan areas are given in Table 1(A) and Figure 3. Forest was the largest land type in the study area during the study period, and was distributed mainly in the middle and low mountainous areas of the extension of Changbai Mountain. The second largest land class was dry land, which mainly located at the alluvial plain areas of Songhua River. The built-up areas, urban and transportation land underwent the rapidest growth (94.04%, 6,739.69 ha and 211.20%, 1,431.82 ha, respectively), rural residence increased by 26.48% (3713.49 ha), isolated industrial and mining land decreased by 29.54% (2,476.14 ha). Built-up land increased 9,408.86 ha during the whole study period. For agricultural and natural land, dry land and paddy fields increased by 15.68% (16,371.72 ha) and 11.78% (4,399.32 ha), forests and orchards decreased by 5.27% (8,143.48 ha) and 4.61% (169.66 ha), respectively, while grasslands almost disappeared during the study period and unused land decreased by 86.04% (20,393.03 ha). In southern China, most of cities have undergone a rapid urban expansion since 1980s, and many agricultural lands were occupied by urban or industrial lands, which has led to a massive loss of agricultural land. However, the intensity of land use in northeastern China is much lower than that in the southern China, and a lot of unused land and some of the forest were developed as agricultural land by farmers to increase their income, which resulted in a slight increase in agricultural land during the study period, even though urban growth occupied some of the agricultural land. Isolated industrial or mining land decreased because some of industries were closed down and the land use changed to residential, commercial or some other type of urban land. Table 1(B, C, D, E) shows the land use changes in the four districts of metropolitan Jilin City. In absolute terms, the greatest urban expansion occurred in Longtan (2,617.97 ha), followed by Fengman (1,739.76 ha), Changyi (1,194.29 ha), Chuanying (1,187.67 ha). In percentage terms, the rapidest urban expansion occurred in Fengman (321.00%), followed by Longtan (125.61%), Chuanying (101.12%), Changyi (35.48%). The urban areas of Fengman district are close to the Songhua Lake, which was a reservoir formed by the construction of Fengman hydroelectric power plant in the upstream segment of Songhua River. A lot of real estate development projects were carried out by the Fengman administrative government due to the good ecological condition, which had led to an extremely rapid urban growth in the last 15 years.

Table 2 shows the land use change matrix in the metropolitan Jilin City. Of the 6,739.69 ha increase in urban land from 1989 to 2005, 3,247.78 ha was changed from isolated industrial and mining land, 1,237.74 ha was changed from dry land, 465.86 ha was changed from forest, and 325.67 ha was changed from paddy fields. Of the 8,380.98 ha isolated industrial or mining land in 1989, 3,247.78 ha was changed to urban land in 2005. Of the 3,713 ha increase in rural residence, 2,730 ha was changed from dry land, 610.37 ha changed from forest. For natural land, 16,862.60 ha forest was transformed to dry land, while grasslands nearly disappeared and were transformed mainly to forest and dry land. Tables 3, 4, 5 and 6 gives the land use change matrix within the four districts. The largest area of land transformed from dry land to urban occurred in Fengman (580.39 ha), followed by Longta (357.14 ha), Chuanying (151.27 ha) and Changyi (148.67 ha). The largest area of land transformed from dry land to rural residence occurred in Chuanying (874.07 ha), followed by Fengman (768.18 ha), Longtan (679.32 ha) and Changyi (408.53 ha). While the greatest area of land transformed from forest to dry land occurred in Longtan (7,610.29 ha) followed by Changyi (4,385.82 ha), Fengman (2,556.86 ha) and Chuanying (2,309.63 ha). The increase of built-up land was mainly transformed from agricultural land, which is a pattern similar to that observed for southern China [9, 10]. A lot of forest was transformed to dry land during the study period, which was also similar to the trend in southern China [9, 10]. Forest is very important for the upstream ecosystem protection of Songhua River, and land use change from forest to dry land may cause soil and water degradation, biodiversity loss and other ecological problems, which will endanger the Songhua River basin.

The land use change analyses in the metropolitan Jilin City and its four districts indicated that: built-up land growth mainly occupied the agricultural land; isolated industrial or mining land decreased moderately; the development of unemployed land to agricultural is very intensive; the transformed area of forest to dry land was tremendous.

### 4.3. Land use changes between dry land and forest on slope land

**Figure 4.**
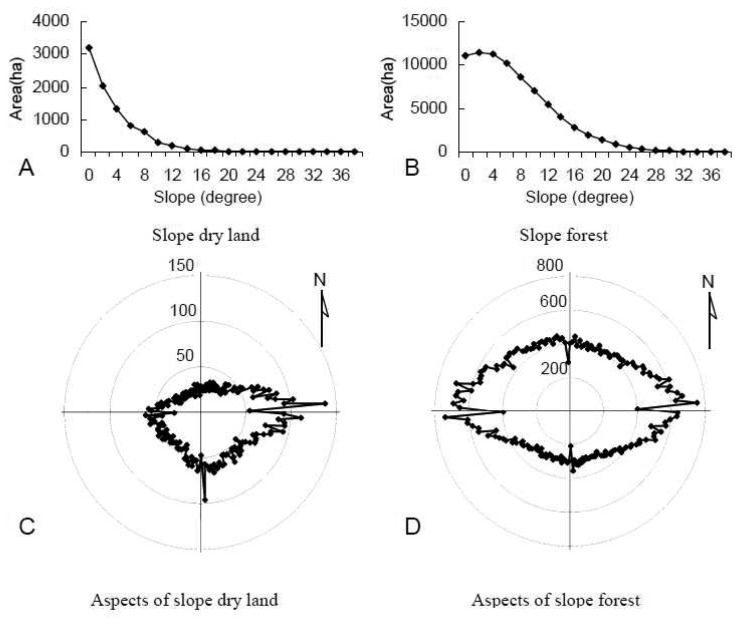
Dry land and forest on sloped land over 4 degrees in 1989.

**Figure 5.**
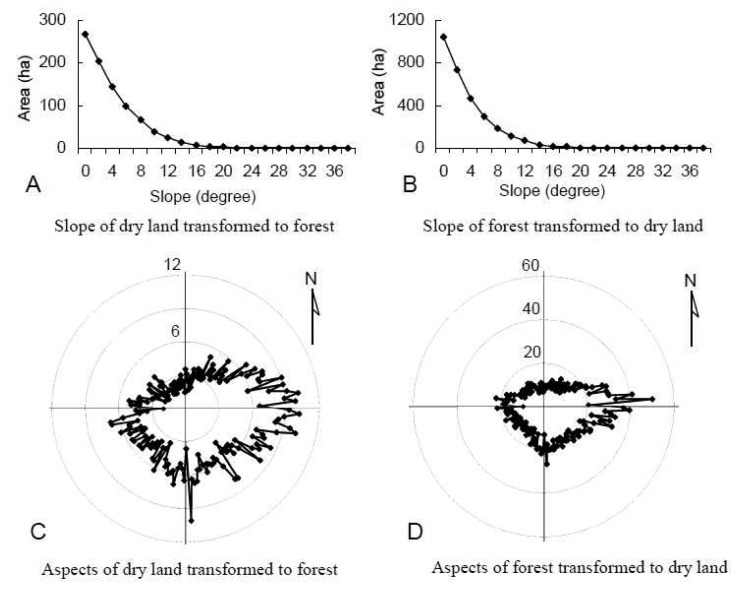
Dry land and forest on sloped land over 4 degrees in 2005.

**Figure 6.**
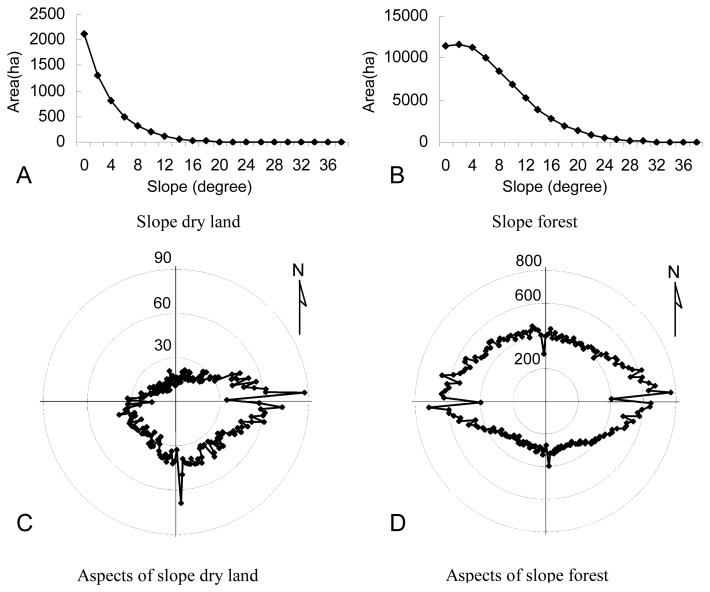
Land transformation between dry land and forest on sloped land over 4 degree.

The two main classes of landscape were dry land and forest, which occupied 68.61% of the study areas in 1989. The slope distribution of dry land and forest land is shown in Table 7. In 1989, dry land in the plain areas was 104,412.39 ha, while its area on sloped land over 4 degrees was 5,543.77 ha, and the dry land on sloped land increased to 8,615.16 ha in 2005. The increase rate of dry land on sloped land was over 55.42%, which was much higher than the increase rate in the plain areas in terms of percentage. Forest on sloped land increased slightly from 77,453.45 to 77,796.64 ha, while the plain area surface decreased strongly from 154,559.02 ha to 146,415.54 ha, most of which were transformed into dry land. Dry land and forest with different slopes and aspects in 1989 and 2005 are shown in Figures 4 and 5, respectively. Sloped dry land was mainly distributed on the land below 10 degrees with an east, southeastern and south sunny direction aspect, and the dry land increase on south and east aspects was higher than that on the other aspects. Forest aspect in 1989 was symmetrically distributed along the south-north axis and east-west axis. Decreased forest was mainly distributed on the south and east aspects with relatively more sunlight. Figure 6 indicates that most of the dry land transformed to forest was located on the east aspect lower than 12 degrees, while forest changed to dry land was mainly distributed on the east and south aspects lower than 10 degrees. Low sloped forest on the east, south, and southeast aspects had a higher transformation probability to dry land. Most of dry land developed on slope land happened before 1998, before the establishment of the policy of Reconverting Cultivated Land into Forest [41]. Figure 6 indicated that on the one hand some of sloped dry lands were transformed into forest again, on the other hand a much larger amount of forest were developed into dry land. Forest protection was still insufficient for the upstream Sonhua River.

### 4.3. Spatial dependency of urban expansion

**Figure 7.**
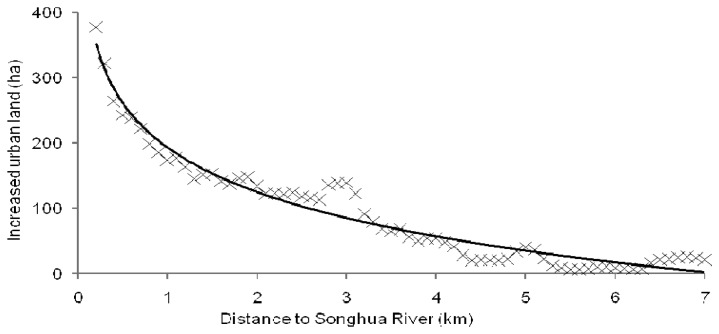
Spatial dependency of land use change from Songhua River between 1989 and 2005

The analysis indicated that urban expansion was strongly dependent upon the distance to the Songhua River (Figure 7). There was a strong distance decay function affecting the increase of urban land. It was obvious that most of urban land increase occurred less than 4 km from the Songhua River. The regression result of the spatial dependency of increased urban land according to the logarithmic model was:

URBincreased=193.0723−98.1346ln(x)

The parameters obtained were: *r*^2^ = 0.91, *F* = 1367, *P* = 0.000, = 0.05, which meant that the regression model was highly significant. The increased urban land declines rapidly in a logarithmic decay function of distance to the Songhua River.

From 1989 to 2005, most of the industries and villages located at Songhua River side in the downtown areas were transformed to urban land, some dry land and paddy fields were transformed into urban land too, which made the urban land increase much faster in the near-river areas with a distance less than 2 km than that in the areas with a distance beyond 5 km.

## 5. Conclusions and Discussion

This paper analyzed the land use changes and the pattern of urban expansion in the metropolitan Jilin City and its four districts from 1989 to 2005, and discussed differences of land use change patterns between southern and northeastern China. The results of this study showed that there had been important land use changes between 1989 and 2003 in the study areas with evidence of urban expansion, forest and grassland degradation. The rapidest urban growth occurred in Fengman district, which is located on the river side of upstream Songhua River. The other three districts experienced similar urban expansions during the study periods. Sloped dry land was mainly distributed on the land below 10 degrees with an east, southeastern and south sunny direction aspect, and most sloped dry land (over 4 degrees) transformed to forest was located with an east aspect lower than 12 degrees, while forest changed to dry land was mainly distributed on east and south aspects lower than 10 degrees. It seemed that low slope forest lower than 10 degrees with east, south, south east aspect had a higher probability of transformation to dry land.

Urban growth in Jilin City occupied a lot of agricultural land and natural land between 1989 and 2005, and this may be attributed to the much higher profits for both farmers and local governments compared to agricultural land use. Most of the agricultural land transformed to urban or other constructive land was high-quality plain land. During the study period the urban land increased nearly 94%, in the meantime, the population increased by 11.5%, GDP increased by 611.3%, and per capita GDP increased by 538% in the study areas, it seems that the economic growth drove the urban land expansion in metropolitan Jilin City. Although some agricultural land was changed to construction land, the total amount of agricultural land did not decrease, which may be attributed to the conversion of forest to agricultural land which compensated the occupation from increased urban land. Transformation from forest to agricultural land occurred in southern China too, which slowed down the loss of agricultural land [22, 23], but this transformation cannot compensate for the occupation by the urban expansion, so the total amount of agricultural land decrease [22, 23]. Land use changes within the agricultural region in southern China was also relatively intensive, especially the transformation of crop land to land type with higher additional value, such as orchards [22, 23]. While in northeastern China, farmers transformed unemployed land and some forest to increase the amount of agricultural land to increase income, land use changes within agricultural land was relatively low intensity. This pattern of agricultural land increase transformed from forest may lead to the forest degradation and threat the ecosystem sustainability in the upstream areas of Songhua River.

Jilin Province has rich forest and water resources, and the protection of forest and river ecosystems was of great importantce not only for the local government, but also for the central government, and land use change analysis around the Changbai Mountain gave some basis for effective river and forest management for the local and central government. During the last two decades, a lot of development zones were established in China. Nearly every city had planned their development projects; even small towns had their own development zones, and many agricultural lands were occupied by these development zones. Jilin City also set up a series of development zones in every district and every county, the five main development zones in metropolitan areas were Jilin New and High Technology Development Zone, Jilin Economic and Technology Development Zone, Chuanying Economic Development Zone, Fengman Economic Development Zone, Longtan Economic Development Zone. According the eleventh five year plan of Jilin City government [42], petrochemistry, automobile, chemical fiber, electric, pharmacy and food processing were the pillar industries encouraged by the local government. Land use has been one of the most important factors that affected the surface water quality within its watershed, and the specific emphasis has been placed on how urbanization negatively influenced air quality [9], surface water quality [6-8], and soil quality [11]. Urbanization in Jilin City is of extreme significance for its sustainable development because most of its urban area is located at the upstream segment of Songhua River. On the 13th November 2005, an explosion happened in a petroleum factory of Jilin Petrochemical Company under China National Petroleum Corporation caused a hundred tons of benzene to flow into the Songhua River, leading directly to the severe pollution of drinking water sources that supplies freshwater to the major cities downstream, including Harbin, capital city of Heilongjiang Province, Khabarovsk of Russia, and other cities in the downstream areas in Russia. This water contamination accident caused by industrial activities showed that urban land development caused by rapid industrialization in Jilin City may lead to great ecological risks for its ecosystems health, especially water quality of Songhua River. The rapid development of the petrochemical and chemical fiber industries need strict measures to control pollution and improve ecosystems management of Songhua River.

## Figures and Tables

**Table 1.** Land use in the metropolitan area of Jilin City between 1989 and 2005 (A: the total metropolitan area; B: Changyi district; C: Chuangying district; D: Fengman district; E: Longtan district).

A	1989		2005		change	

	Area (ha)	Area (%)	Area (ha)	Area (%)	Area (ha)	Area (%)
	
URB	7166.6	1.9	13906.29	3.68	6739.69	94.04
IIM	8380.98	2.22	5904.84	1.56	-2476.14	-29.54
RR	14025.94	3.72	17739.43	4.7	3713.49	26.48
TRN	677.93	0.18	2109.75	0.56	1431.82	211.2
DL	104412.4	27.66	120784.1	32	16371.72	15.68
PAD	37331.82	9.89	41731.14	11.06	4399.32	11.78
FOR	154559	40.95	146415.5	38.79	-8143.48	-5.27
ORC	3677.64	0.97	3507.98	0.93	-169.66	-4.61
GRS	1554	0.41	13.73	0	-1540.27	-99.12
WAT	21974.9	5.82	22041.44	5.84	66.54	0.3
UNE	23700.72	6.28	3307.69	0.88	-20393	-86.04

B	1989		2005		change	

	Area (ha)	Area (%)	Area (ha)	Area (%)	Area (ha)	Area (%)
	
URB	3365.98	3.93	4560.27	5.32	1194.29	35.48
IIM	2221.05	2.59	1537.21	1.79	-683.84	-30.79
RR	4263.31	4.97	4881.79	5.7	618.48	14.51
TRN	278.72	0.33	433.66	0.51	154.94	55.59
DL	30210.5	35.25	33954.48	39.62	3743.98	12.39
PAD	15734.52	18.36	17449.9	20.36	1715.38	10.9
FOR	21956.03	25.62	18836.03	21.98	-3120	-14.21
ORC	355.2	0.41	332.64	0.39	-22.56	-6.35
GRS	7.81	0.01	13	0.02	5.19	66.45
WAT	3365.98	3.93	4560.27	5.32	1194.29	35.48
UNE	2221.05	2.59	1537.21	1.79	-683.84	-30.79

C	1989		2005		change	

	Area (ha)	Area (%)	Area (ha)	Area (%)	Area (ha)	Area (%)
	
URB	1174.48	1.71	2362.15	3.45	1187.67	101.12
IIM	1316.11	1.92	1137	1.66	-179.11	-13.61
RR	3214.1	4.69	4269.41	6.23	1055.31	32.83
TRN	20.49	0.03	394.75	0.58	374.26	1826.55
DL	25740.12	37.54	28435.31	41.47	2695.19	10.47
PAD	6757.47	9.86	7867.33	11.47	1109.86	16.42
FOR	20769.62	30.29	20390.83	29.74	-378.79	-1.82
ORC	1414.17	2.06	1430.63	2.09	16.46	1.16
GRS	796.59	1.16	0.3	0	-796.29	-99.96
WAT	1481.81	2.16	1597.63	2.33	115.82	7.82
UNE	5878.85	8.57	678.47	0.99	-5200.38	-88.46

D	1989		2005		change	

	Area (ha)	Area (%)	Area (ha)	Area (%)	Area (ha)	Area (%)
	
URB	541.98	0.51	2281.74	2.15	1739.76	321
IIM	1020.59	0.96	1021.7	0.96	1.11	0.11
RR	2142.55	2.02	3499.97	3.3	1357.42	63.36
TRN	124.47	0.12	297.03	0.28	172.56	138.64
DL	14799.34	13.95	16886.54	15.92	2087.2	14.1
PAD	2322.76	2.19	2599.64	2.45	276.88	11.92
FOR	64277.16	60.58	65052.09	61.31	774.93	1.21
ORC	736.3	0.69	992.71	0.94	256.41	34.82
GRS	536.6	0.51	0.43	0	-536.17	-99.92
WAT	12925.59	12.18	12693.12	11.96	-232.47	-1.8
UNE	6667.97	6.28	770.34	0.73	-5897.63	-88.45

E	1989		2005		change	

	Area (ha)	Area (%)	Area (ha)	Area (%)	Area (ha)	Area (%)
	
URB	2084.16	1.78	4702.13	4.02	2617.97	125.61
IIM	3823.23	3.27	2208.93	1.89	-1614.3	-42.22
RR	4405.98	3.76	5088.26	4.35	682.28	15.49
TRN	254.25	0.22	984.31	0.84	730.06	287.14
DL	33662.43	28.75	41507.78	35.45	7845.35	23.31
PAD	12517.07	10.69	13814.27	11.8	1297.2	10.36
FOR	47556.21	40.61	42136.59	35.99	-5419.62	-11.4
ORC	1171.97	1	752	0.64	-419.97	-35.83
GRS	213	0.18	0	0	-213	-100
WAT	4429.62	3.78	4782.74	4.08	353.12	7.97
UNE	6973.84	5.96	1114.75	0.95	-5859.09	-84.02

**Table 2.** Land use change matrix in the metropolitan area of Jilin City between 1989 and 2005.

	URB	IIM	RR	TRN	DL	PAD	FOR	ORC	GRS	WAT	UNE	1989
	
URB	7122.77	0.00	0.00	43.06	0.00	0.00	0.00	0.00	0.00	0.00	0.77	7166.60
IIM	3247.78	2879.25	287.61	61.15	350.21	455.45	538.75	10.11	0.00	382.22	168.45	8380.98
RR	869.22	255.26	12752.20	149.24	0.00	0.00	0.00	0.00	0.00	0.00	0.00	14025.94
TRN	114.17	5.02	17.30	442.63	40.16	29.79	20.25	0.39	0.00	0.98	7.24	677.93
DL	1237.47	673.75	2730.10	509.70	90972.77	2936.36	3508.70	738.17	0.02	700.30	405.05	104412.39
PAD	325.67	549.43	383.00	325.37	0.00	34808.85	311.84	34.75	5.45	465.56	121.90	37331.82
FOR	465.86	760.49	610.37	251.02	16862.60	769.91	131914.70	818.13	0.28	1134.54	971.12	154559.02
ORC	58.01	43.33	98.17	17.01	658.89	11.90	1375.95	1327.28	0.05	25.99	61.06	3677.64
GRS	0.16	9.15	20.52	0.00	448.75	19.71	964.23	22.61	7.48	23.83	37.56	1554.00
WAT	173.20	175.12	180.31	79.56	1341.17	1185.74	558.70	11.60	0.07	18123.65	145.78	21974.90
UNE	291.98	554.04	659.83	231.01	10109.56	1513.43	7222.42	544.94	0.38	1184.37	1388.76	23700.72
2005	13906.30	5904.84	17739.4	2109.75	120784.11	41731.14	146415.54	3507.98	13.73	22041.44	3307.69	377461.94

**Table 3.** Land use change matrix in Changyi district between 1989 and 2005

	URB	IIM	RR	TRN	DL	PAD	FOR	ORC	GRS	WAT	UNE	1989
	
URB	3353.68	0.00	0.00	12.00	0.00	0.00	0.00	0.00	0.00	0.00	0.30	3365.98
IIM	644.49	824.58	77.00	8.01	68.69	429.33	36.74	0.37	0.00	122.45	9.39	2221.05
RR	73.03	36.80	4133.63	19.85	0.00	0.00	0.00	0.00	0.00	0.00	0.00	4263.31
TRN	4.69	0.63	1.93	241.51	3.40	18.6	6.58	0.00	0.00	0.50	0.88	278.72
DL	148.67	113.38	408.53	43.49	27275.60	1251.03	700.83	12.05	0.00	219.30	37.64	30210.50
PAD	274.57	418.35	109.93	54.65	0.00	14651.20	64.88	0.01	5.45	144.79	10.70	15734.52
FOR	8.93	74.12	53.44	2.61	4385.82	127.81	17072.03	21.76	0.00	125.40	84.11	21956.03
ORC	20.37	2.83	3.83	0.51	19.61	1.39	12.46	293.95	0.00	0.00	0.25	355.20
GRS	0.00	0.00	0.00	0.00	0.32	0.00	0.01	0.00	7.48	0.00	0.00	7.81
WAT	19.48	20.85	37.92	21.72	348.05	495.68	52.03	0.08	0.07	2122.10	19.86	3137.88
UNE	12.36	45.67	55.58	29.31	1853.01	474.87	890.47	4.42	0.00	233.37	581.00	4180.06
2005	4560.27	1537.21	4881.79	433.66	33954.50	17449.9	18836.03	332.64	13.00	2968.00	744.13	85711.06

**Table 4.** Land use change matrix in Chuanying district between 1989 and 2005

	URB	IIM	RR	TRN	DL	PAD	FOR	ORC	GRS	WAT	UNE	1989
	
URB	1171.41	0.00	0.00	2.88	0.00	0.00	0.00	0.00	0.00	0.00	0.19	1174.48
IIM	627.26	377.99	88.36	8.31	120.76	6.02	40.31	2.10	0.00	4.56	40.44	1316.11
RR	308.35	56.9	2821.55	27.30	0.00	0.00	0.00	0.00	0.00	0.00	0.00	3214.10
TRN	0.49	0.84	0.88	6.82	6.41	0.32	2.92	0.20	0.00	0.02	1.59	20.49
DL	151.27	230.91	874.07	178.14	22360.52	591.25	803.43	331.32	0.02	100.21	118.98	25740.12
PAD	6.70	36.03	92.70	63.92	0.00	6395.19	52.64	7.28	0.00	84.71	18.30	6757.47
FOR	28.69	229.33	120.32	45.82	2309.63	123.15	17348.60	243.09	0.28	127.06	193.70	20769.62
ORC	8.69	21.58	48.38	3.21	208.23	2.57	443.56	638.15	0.00	5.14	34.66	1414.17
GRS	0.01	5.89	12.86	0.00	256.86	16.84	449.94	19.21	0.00	14.62	20.36	796.59
WAT	21.19	14.36	50.34	4.03	208.24	205.30	39.98	2.00	0.00	919.17	17.20	1481.81
UNE	38.09	163.17	159.95	54.32	2964.66	526.69	1209.50	187.28	0.00	342.14	233.05	5878.85
2005	2362.15	1137.00	4269.41	394.75	28435.31	7867.33	20390.80	1430.60	0.30	1597.63	678.47	68563.81

**Table 5.** Land use change matrix in Fengman district between 1989 and 2005

	URB	IIM	RR	TRN	DL	PAD	FOR	ORC	GRS	WAT	UNE	1989
	
URB	541.93	0.00	0.00	0.00	0.00	0.00	0.00	0.00	0.00	0.00	0.05	541.98
IIM	468.05	253.53	68.48	10.78	51.21	1.51	81.68	5.32	0.00	22.65	57.38	1020.59
RR	143.22	43.42	1934.32	21.59	0.00	0.00	0.00	0.00	0.00	0.00	0.00	2142.55
TRN	25.83	3.40	7.98	57.93	11.85	4.78	8.73	0.19	0.00	0.27	3.51	124.47
DL	580.39	168.75	768.18	74.46	11491.77	221.39	998.95	276.88	0.00	104.32	114.25	14799.34
PAD	3.04	37.36	59.58	22.73	0.00	2065.42	55.92	19.08	0.00	40.14	19.49	2322.76
FOR	303.36	239.45	257.32	29.93	2556.86	100.87	59985.55	181.43	0.00	387.52	234.87	64277.16
ORC	17.73	7.45	31.58	6.60	219.09	3.34	174.08	245.64	0.05	11.82	18.92	736.30
GRS	0.15	3.26	7.59	0.00	155.56	1.31	351.39	3.40	0.00	8.86	5.08	536.60
WAT	79.31	82.56	53.48	29.65	229.03	80.67	345.02	7.64	0.00	11979.2	39.04	12925.59
UNE	118.73	182.52	311.46	43.36	2171.17	120.35	3050.77	253.13	0.38	138.35	277.75	6667.97
2005	2281.74	1021.7	3499.97	297.03	16886.54	2599.64	65052.09	992.71	0.43	12693.10	770.34	106095.31

**Table 6.** Land use change matrix in Longtan district between 1989 and 2005

	1989	Plain		%	Slope	%	2005	Plain		%	Slope	%
	
	URB	IIM	RR	TRN	DL	PAD	FOR	ORC	GRS	WAT	UNE	1989
URB	2055.75	0.00	0.00	28.18	0.00	0.00	0.00	0.00	0.00	0.00	0.23	2084.16
IIM	1507.98	1423.15	53.77	34.05	109.55	18.59	380.02	2.32	0.00	232.56	61.24	3823.23
RR	344.62	118.14	3862.72	80.50	0.00	0.00	0.00	0.00	0.00	0.00	0.00	4405.98
TRN	83.16	0.15	6.51	136.37	18.50	6.09	2.02	0.00	0.00	0.19	1.26	254.25
DL	357.14	160.71	679.32	213.61	29844.9	872.69	1005.49	117.92	0.00	276.47	134.18	33662.43
PAD	41.36	57.69	120.79	184.07	0.00	11697.1	138.4	8.38	0.00	195.92	73.41	12517.07
FOR	124.88	217.59	179.29	172.66	7610.29	418.08	37508.57	371.85	0.00	494.56	458.44	47556.21
ORC	11.22	11.47	14.38	6.69	211.96	4.60	745.85	149.54	0.00	9.03	7.23	1171.97
GRS	0.00	0.00	0.07	0.00	36.01	1.56	162.89	0.00	0.00	0.35	12.12	213.00
WAT	53.22	57.35	38.57	24.16	555.85	404.09	121.67	1.88	0.00	3103.15	69.68	4429.62
UNE	122.80	162.68	132.84	104.02	3120.72	391.52	2071.68	100.11	0.00	470.51	296.96	6973.84
2005	4702.13	2208.93	5088.26	984.31	41507.78	13814.3	42136.59	752	0.00	4782.74	1114.75	117091.76

**Table 7.** Area of dry land and forest distributed on the plain and slope region (over 4 degrees)

DL	104412.39	98868.62	94.69	5543.77	5.31	120784.11	112168.95	92.87	8615.16	7.13
FOR	154559.02	77453.45	50.11	77105.57	49.89	146415.54	68618.90	46.87	77796.64	53.13

## References

[1] Vitousek P.M. (1994). Beyond global warming: ecology and global change. Ecology.

[2] Taniguchi M., Uemura T. (2005). Effects of urbanization and groundwater flow on the subsurface temperature in Osaka, Japan. Phys. Earth Planet. Int..

[3] Chalmers A.T., Van Metre P.C., Callender E. (2007). The chemical response of particle-associated contaminants in aquatic sediments to urbanization in New England, U.S.A. J. Contam. Hydrol..

[4] Zilberbrand M., Rosenthal E., Shachnai E. (2001). Impact of urbanization on hydrochemical evolution of groundwater and on unsaturated-zone gas composition in the coastal city of Tel Aviv, Israel. J. Contam. Hydrol..

[5] Al-Kharabsheh A.A. (1999). Influence of urbanization on water quality at Wadi Kufranja Basin (Jordan). J. Arid Environ..

[6] Rose S. (2007). The effects of urbanization on the hydrochemistry of base flow within the Chattahoochee River Basin (Georgia, USA). J. Hydrol..

[7] Berry Lyons W., Fitzgibbon T.O., Welch K.A., Carey A. E. (2006). Mercury geochemistry of the Scioto River, Ohio: Impact of agriculture and urbanization. Appl. Geochemistry.

[8] Jeong C.H. (2001). Effect of land use and urbanization on hydrochemistry and contamination of groundwater from Taejon area, Korea. J. Hydrol..

[9] Civerolo K., Hogrefe C., Lynn B., Rosenthal J., Ku J., Solecki W., Cox J., Small C., Rosenzweig C., Goldberg R., Knowlton K., Kinney P. (2007). Estimating the effects of increased urbanization on surface meteorology and ozone concentrations in the New York City metropolitan region. Atmos. Environ..

[10] Yamada T. (1999). A numerical simulation of urbanization on the local climate. J. Wind Eng. Ind. Aerod..

[11] Pauchard A., Aguayo M., Peña E., Urrutia R. (2006). Multiple effects of urbanization on the biodiversity of developing countries: The case of a fast-growing metropolitan area (Concepción, Chile). Biol. Conserv..

[12] Alig R.J., Kline J.D., Lichtenstein M. (2004). Urbanization on the US landscape: looking ahead in the 21st century. Landscape Urban Plan..

[13] Hathout S. (2002). The use of GIS for monitoring and predicting urban growth in East and West St Paul, Winnipeg, Manitoba, Canada. J. Environ. Manage..

[14] Jensen J.R., Cowen D.C. (1999). Remote sensing of urban suburban infrastructure and socio-economic attributes. Photogramm. Eng. Rem. S..

[15] Ningal T., Hartemink A.E., Bregt A.K. Land use change and population growth in the Morobe Province of Papua New Guinea between 1975 and 2000. J. Environ. Manage..

[16] Shajaat Ali A.M. (2006). Rice to shrimp: Land use/land cover changes and soil degradation in Southwestern Bangladesh. Land Use Policy.

[17] Seto K.C., Kaufmann R.K. (2003). Modelling the drivers of urban land use change in the Pearl River Delta, China: integrating remote sensing with socioeconomic data. Land Econ..

[18] Ren W., Zhong Y., Meligrana J., Anderson B., Edgar Watt W., Chen J., Leung H. (2003). Urbanization, land use, and water quality in Shanghai 1947–1996. Environ. Int..

[19] Zhao B., Kreuter U., Li Bo., Ma Z., Chen J., Nakagoshi N. (2004). An ecosystem service value assessment of land-use change on Chongming Island, China. Land Use Policy.

[20] Long H., Tang G., Li X., Heilig G.K. (2007). Socio-economic driving forces of land-use change in Kunshan, the Yangtze River Delta economic area of China. J. Environ. Manage..

[21] Xie Y., Yu M., Bai Y., Xing X. (2006). Ecological analysis of an emerging urban landscape pattern— desakota: a case study in Suzhou, China. Landscape ecol..

[22] Weng Q.H. (2002). Land use change analysis in the Zhujiang Delta of China using satellite remote sensing, GIS and stochastic modelling. J. Environ. Manage..

[23] Fan F., Weng Q., Wang Y. (2007). Land Use and Land Cover Change in Guangzhou, China, from 1998 to 2003, Based on Landsat TM /ETM+ Imagery. Sensors.

[24] Qian L., Cut H., Chang J. (2006). Impacts of Land Use and Cover Change on Land Surface Temperature in the Zhujiang Delta. Pedosphere.

[25] Li X., Yeh A.G. (2004). Analyzing spatial restructuring of land use patterns in a fast growing region using remote sensing and GIS. Landscape Urban Plan..

[26] Li X., Yeh A.G. (1998). Principal component analysis of stacked multitemporal images for the monitoring of rapid urban expansion in the Pearl River Delta. Int. J. Remote. Sens..

[27] Quan B., Chen J., Qiu H., ROMKENS M.J.M., Yang X., Jiang S., Li B. (2006). Spatial-Temporal Pattern and Driving Forces of Land Use Changes in Xiamen. Pedosphere.

[28] Chen J., Wei S., Chang K., Tsai B. (2007). A comparative case study of cultivated land changes in Fujian and Taiwan. Land Use Policy.

[29] Cao W., Zhu H., Chen S. (2007). Impacts of urbanization on topsoil nutrient balances—a case study at a provincial scale from Fujian, China. CATENA.

[30] Xinhua News Agency Northeastern China was expected to be the fourth economic increase pole. http://news.xinhuanet.com/fortune/2006-09/26/content_5140003.htm.

[31] Consolidation patterns and emperies of slope black cropland for the Northeastern China. http://www.swcc.org.cn/zhuanti2_view_content.asp?id=15880.

[32] Lunetta R.S., Knight J.F., Ediriwickrema J., Lyon J.G., Worthy L.D. (2006). Land-cover change detection using multi-temporal MODIS NDVI data. Remote Sens. Environ..

[33] Xiao H., Weng Q. (2007). The impact of land use and land cover changes on land surface temperature in a karst area of China. J. Environ. Manage..

[34] Turner M.G. (1990). Landscape changes in nine rural counties in Georgia. Photogramm. Eng. Rem. S..

[35] Harris P.M., Ventura S.J. (1995). The integration of geographic data with remotely sensed imagery to improve classification in an urban area. Photogramm. Eng. Rem. S..

[36] Assali S., Menenti M. (2000). Mapping vegetation–soil–climate complexes in southern Africa using temporal Fourier analysis of NOAA-AVHRR NDVI data. Int. J. Remote Sens..

[37] Homer C., Huang C., Limin Y., Wylie B., Coan M. (2004). Development of a 2001 national land cover database for the United States. Photogramm. Eng. Rem. S..

[38] Knight J.K., Lunetta R.L., Ediriwickrema J., Khorram S. (2006). Regional scale land-cover characterization using MODIS-NDVI 250 m multi-temporal imagery: A phenology based approach. GIScience and Remote Sensing (Special Issue on Multi-Temporal Imagery Analysis).

[39] Loveland T.R., Merchant J.W., Ohlen D.O., Brown J.F. (1991). Development of a land cover characteristics data base for the conterminous U.S.. Photogramm. Eng. Rem. S..

[40] Lambin E.F., Ehrlich D. (1997). Land-cover changes in Sub-Saharan Africa (1982–1991): Application of a change index based on remotely sensed surface temperature and vegetation indices at a continental scale. Remote Sens. Environ..

[41] People's government of Jilin municipality The 11^th^ five year social and economic development plan of Jilin City. http://www.jlcity.gov.cn/jl_577/infocontent.jsp?infoid=6730.

[42] Development and Reform Commission of Jilin City Guide lines of the 10^th^ five year social and economic development plan of Jilin City. http://www.jlsdrc.gov.cn/fazhan/.

